# Oral compound probiotic supplements can improve the quality of life for patients with lung cancer during chemotherapy: A randomized placebo‐controlled study

**DOI:** 10.1111/1759-7714.15177

**Published:** 2023-11-29

**Authors:** Hao Wei, Zhiying Yue, Jialong Han, Ping Chen, Ke Xie, Yu Sun, Jiang Zhu

**Affiliations:** ^1^ Thoracic Oncology Ward, Cancer Center, West China Hospital Sichuan University Chengdu China; ^2^ Department of Biotherapy, Cancer Center, West China Hospital Sichuan University Chengdu China; ^3^ Cancer Center Chengdu China; ^4^ Cancer Center, Sichuan Academy of Medical Sciences & Sichuan Provincial People's Hospital, School of Medicine University of Electronic Science and Technology of China Chengdu China; ^5^ Radiotherapy Physics & Technology Center, Cancer Center, West China Hospital Sichuan University Chengdu China; ^6^ Department of Medical Oncology, Shangjin branch of West China Hospital Sichuan University Chengdu China

**Keywords:** chemotherapy, gut microbiota, lung cancer, probiotics, quality of life

## Abstract

**Background:**

Chemotherapy is an important approach for lung cancer patients. The study was designed to evaluate the feasibility of the compound probiotic supplements in improving the quality of life for lung cancer patients undergoing chemotherapy.

**Methods:**

This randomized, double‐blind, placebo‐controlled trial enrolled chemotherapy‐naive patients with lung cancer who were scheduled to receive platinum‐based doublet chemotherapy. All eligible patients were randomly administered (1:1) compound probiotic supplements (group BP‐1) or placebo (group C) for two chemotherapy cycles. The EORTC QLQ C30 questionnaire scores were evaluated before the first, second, and third cycles of chemotherapy. The primary endpoint was the difference in the EROTC QLQ C30 questionnaire score between the two groups after two cycles of chemotherapy.

**Results:**

A total of 110 patients were recruited from March 2021 to January 2022. After undergoing two cycles of chemotherapy, group BP‐1 were significantly better in various dimensions of the overall quality of life, role function, nausea and vomiting, appetite loss, constipation, and diarrhea relative to group C (76.90 ± 18.31 vs. 58.89 ± 17.17; 93.33 ± 11.58 vs. 85.93 ± 15.06; 0.00 ± 0.00 vs. 27.04 ± 29.15; 6.67 ± 13.53 vs. 22.22 ± 18.80; 0.95 ± 5.63 vs. 28.15 ± 22.42; 2.86 ± 9.47 vs. 15.56 ± 16.82; *p* < 0.05, respectively). The incidence of nausea and vomiting, appetite loss, constipation, and diarrhea in group BP‐1 was significantly lower than in group C (0% vs. 71.43%, 16.67% vs. 57.14%, 2.38% vs. 63.27%, and 7.14% vs. 42.86%, respectively, *p* < 0.001).

**Conclusions:**

Compound probiotic supplements can improve the quality of life and relieve chemotherapy‐related gastrointestinal side effects for lung cancer patients receiving platinum‐based doublet chemotherapy. (Chinese Clinical Trial Registry: ChiCTR1800019269).

## Graphical abstract

Our study demonstrated that oral compound probiotics supplements can improve the quality of life and relieve chemotherapy‐related gastrointestinal adverse events for lung cancer patients receiving platinum‐based chemotherapy.

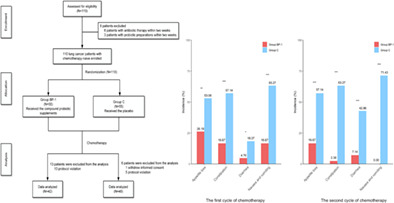

## INTRODUCTION

Despite significant improvements in the realm of anticancer strategies, whatever chemotherapy is administered as a monotherapy or in combination with other treatment paradigms encompassing surgery, radiotherapy, immune checkpoint inhibitors (ICIs), antiangiogenesis agents, targeted therapies, and beyond, it remains an important treatment strategy for almost all cancers, especially lung cancer, the most prevalent and fatal malignant tumor in the population.^1^, ^2^

Platinum‐based doublet chemotherapy occupies an important role in the management of lung cancer; however, there are treatment‐associated side effects which include nausea and vomiting, fatigue, appetite loss, pain, constipation, diarrhea, and many other debilitating symptoms that may deteriorate the quality of life for patients undergoing chemotherapy, and even necessitate dose reduction or discontinuation.^3^, ^4^

Chemotherapy can also damage the gastrointestinal epithelial cells and result in a disorder of the gut microbiome.^5^, ^6^ Gut microbiota has been proven to protect the intestinal mucosa, prevent intestinal inflammation, and build the immune ecology of the whole body.^7^, ^8^ Previous studies showed that certain cytotoxic drugs such as cyclophosphamide, fluorouracil, and etoposide appeared to have antibacterial properties in plasma, and medications such as irinotecan, fluorouracil would affect the diversity of the gut microbiome,^9^, ^10^ which may result in gastrointestinal mucositis, exacerbating the mucositis caused by chemotherapeutic drugs, thereby leading to severe gastrointestinal complications in patients receiving chemotherapy.^11^, ^12^, ^13^, ^14^

Many strategies for controlling chemotherapy‐related adverse events have been applied in clinical practice during the past decades, but the management situation is not optimistic in the real world.^3^, ^15^, ^16^ Emerging evidence favors the strategy of gut microbiota regulation for ameliorating chemotherapy‐related adverse events.^17^ In particular, the feasibility of probiotic supplementation to ameliorate chemotherapy‐related adverse effects has been demonstrated in preclinical and clinical studies.^18^, ^19^, ^20^, ^21^, ^22^, ^23^, ^24^, ^25^ Nevertheless, whether administering compound probiotics can relieve chemotherapy‐related adverse events for lung patients undergoing platinum‐based doublet chemotherapy is rarely reported. This study attempted to determine whether oral compound probiotic supplements can reduce chemotherapy‐related adverse effects and improve lung cancer patients quality of life during chemotherapy.

## METHODS

This prospective, randomized, placebo‐controlled, multicenter clinical study was conducted at three major cancer centers in Sichuan province, China: Thoracic Oncology Ward, West China Hospital, Sichuan University; Cancer Center, No.7 People's Hospital of Chengdu; Cancer Center, People's Hospital of Sichuan Province. The study was approved by the Ethics Committee of the West China Hospital of Sichuan University and conformed to the Declaration of Helsinki. The study was registered in the Chinese Clinical Trial Registry (registration no.: ChiCTR1800019269). All eligible patients provided written informed consent.

### Patients

**FIGURE 1:**
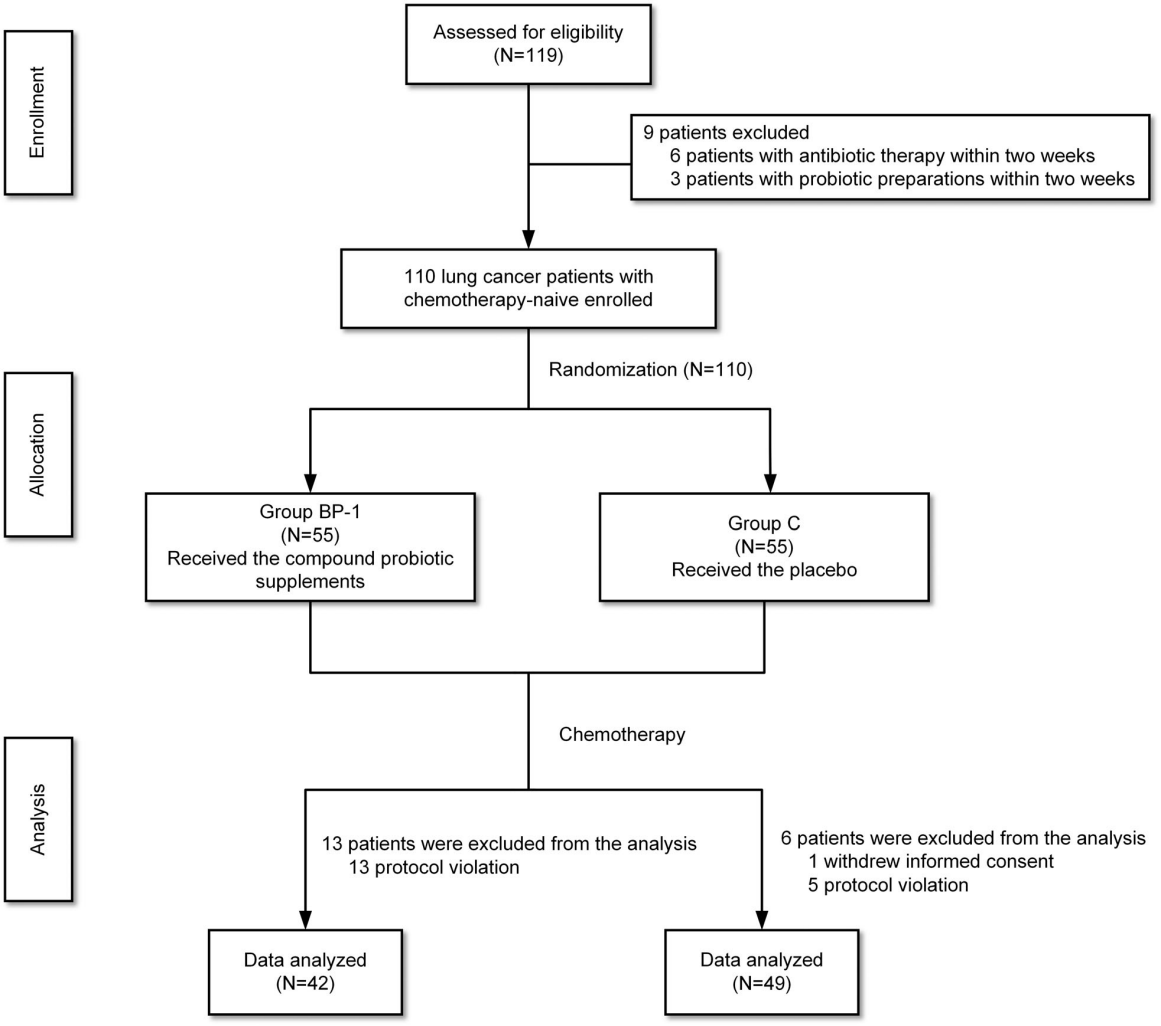
The flow chart of patient disposition.

This study intended to screen and enroll chemotherapy‐naive patients with lung cancer who were scheduled to receive platinum‐based doublet chemotherapy. The quality of life score of lung cancer patients during chemotherapy was around 60 (Figure 1). It was expected that it could be improved to 70 by using a compound probiotic preparation, taking the test level α as 0.05, assuming that the number of cases in both groups was equal, the minimum sample size to be included in each group was n1 = n2 = 49, *N* = 98, considering 10% of patients were excluded, the final number of cases would be 110.

Patients who met all of the following criteria were included: (1) pathologically confirmed with lung cancer (including non‐small cell lung cancer and small cell lung cancer); (2) chemotherapy‐naive; (3) aged between 18 and 75 years old; (4) Eastern Cooperative Oncology Group performance status (ECOG PS) 0–2; (5) receiving chemotherapy for the first time with a regimen of cisplatin/carboplatin (AUC ≥5) in combination with one of these following agents: paclitaxel, docetaxel, gemcitabine, vinorebine, pemetrexed, etoposide, and who were supposed to receive two or more cycles of chemotherapy.

The main exclusion criteria were as follows: (1) Patients who received recent (within 2 weeks before chemotherapy) antibiotic therapy or proton pump inhibitor therapy, (2) patients who had recently (within 2 weeks before chemotherapy) taken some probiotic preparations, (3) patients with chronic gastrointestinal diseases or gastrointestinal metastatic tumor, (4) patients with severe systemic metabolic diseases or immune system diseases.

Based on the computer‐generated program, enrolled patients were randomly assigned (1:1) to either the compound probiotic supplements group (group BP‐1) or the placebo group (group C). A total of 110 random identification numbers were created by the computer before patients enrolled. Each enrolled patient was given an identification number based on the enrolment order and assigned to the corresponding group based on the identification number. The process mentioned above was performed by the Cancer Psychology and Health Management Committee of the Sichuan Cancer Society and was double‐blind for both subjects and researchers.

### Treatment

The enrolled patients were administered the platinum‐based doublet chemotherapy regimen recommended by NCCN/CSCO guidelines.^26^

The oral compound probiotic supplements (Hua Wei Yi probiotic solid drink, Yiga Bio‐technology Chengdu Co., LTD, Chengdu, China) contained oligofructose (added at >93.69%), *Bifidobacterium lactis Bi‐07, Lactobacillus acidophilus NCFM, Lactobacillus rhamnosus HN001, Bifidobacterium lactis HN019*. It was packaged in an aluminum‐plastic film bag and a maltodextrin‐based placebo with the same appearance and taste. All compound probiotics/placebos in this study were received from Yiga Bio‐technology Chengdu Co., Ltd (via the Cancer Psychology and Health Management Committee of the Sichuan Cancer Society. The corresponding compound probiotic supplement/placebo with an identification number was assigned when a patient was enrolled. The Cancer Psychology and Health Management Committee of the Sichuan Cancer Society kept the assignment information list and was responsible for compound probiotic supplement/placebo distribution. The enrolled patients were given compound probiotic supplements/placebos at the beginning of the first cycle of chemotherapy, one sachet (2 g) dissolved in cold water or indoor temperature twice a day until the start of the third chemotherapy cycle.

### Follow‐up and data collection

All enrolled patients were evaluated for two chemotherapy cycles. The EORTC QLQ C30 questionnaire was completed independently by the enrolled patients prior to the first, second, and third cycles of chemotherapy. The scale consisted of 30 items, including five functional dimensions (physical function, role function, cognitive function, emotional function, and social function), nine symptom dimensions (fatigue, pain, nausea and vomiting, dyspnea, insomnia, appetite loss, constipation, diarrhea, financial difficulties), and one dimension of overall quality of life.^27^ For the functional and overall quality of life dimensions, higher scores indicated better functional status and quality of life. In contrast, for the symptom dimensions, higher scores indicated more severe symptoms or problems.

The National Cancer Institute Common Toxicity Criteria (version 4.0) was used for the evaluation of adverse events.

### Statistical analysis

The primary endpoint was the difference in the EROTC QLQ C30 questionnaire score between the two groups after two cycles of chemotherapy. For qualitative data, we used the chi‐square test to determine whether there was a difference between groups BP‐1 and C. The Mann–Whitney U or independent‐sample *t*‐test were used to compare the differences for quantitative data. The above statistical analysis was performed with SPSS version 27 software. Two‐sided *p*‐values < 0.05 were determined to be statistically significant.

## RESULTS

### Patient characteristics

From March 2021 to January 2022, 110 patients were enrolled, of whom one patient withdrew their informed consent and 18 patients were excluded due to protocol violation. A total of 91 patients were included in the final statistics. No significant differences were observed between the two groups at baseline (Table 1).

**TABLE 1:** Baseline demographic characteristics of 91 lung cancer patients.

Characteristic	Group C (*N* = 49)	Group BP‐1 (*N* = 42)	*p*‐value
Age (year), mean (SD)	60.06 (7.67)	58.95 (8.51)	0.515
Gender, *n* (%)			
Male	33 (67.35)	30 (71.43)	0.674
Female	16 (32.65)	12 (28.57)	
Ethnicity, *n* (%)			
Han	48 (97.96)	41 (97.62)	1.000
Other	1 (2.04)	1 (2.38)	
History of allergy, *n* (%)			
No	49 (100.00)	42 (100.00)	‐
History of significant past, *n* (%)			
No	49 (100.00)	42 (100.00)	‐
Stage, *n* (%)			0.249
I	12 (24.49)	6 (14.29)	
II	8 (16.33)	14 (33.33)	
III	19 (38.78)	15 (35.71)	
IV	10 (20.41)	7 (16.67)	
History of surgery, *n* (%)			
Yes	27 (55.10)	25 (59.52)	0.671
No	22 (44.90)	17 (40.48)	
Performance status, *n* (%)			
0	40 (81.63)	32 (76.19)	0.524
1	9 (18.37)	10 (23.81)	
Chemotherapy regimen			
Pemetrexed + carboplatin	19 (38.78)	16 (38.10)	0.680
Pemetrexed + cisplatinum	6 (12.24)	7 (16.67)	
Etoposide + cisplatinum	5 (10.20)	7 (16.67)	
Gemcitabine + cisplatinum	2 (4.08)	1 (2.38)	
Paclitaxel + carboplatin	7 (14.29)	7 (16.67)	
Paclitaxel + cisplatinum	10 (20.41)	4 (9.52)	

Abbreviations: SD, standard deviation.

### EORTC QLQ C30 questionnaire analysis

No significant difference was observed in the scores of the EORTC QLQ C30 questionnaire at baseline between the two groups (Table 2).

**TABLE 2:** Quality of life before and after receiving interventions and chemotherapy treatment in different groups.

Dimensions Mean (SD)	Group C (*N* = 49)	Group BP‐1 (*N* = 42)	*p*‐value
Overall quality of life			
Before the first cycle of chemotherapy	67.21 (23.92)	73.20 (19.56)	0.304
Before the second cycle of chemotherapy	61.55 (19.12)	75.45 (15.46)	0.001
Before the third cycle of chemotherapy	58.89 (17.17)	76.90 (18.31)	<0.001
Functional dimensions			
Physical function			
Before the first cycle of chemotherapy	87.83 (13.36)	85.23 (10.67)	0.085
Before the second cycle of chemotherapy	89.24 (8.41)	88.11 (10.08)	0.666
Before the third cycle of chemotherapy	90.96 (8.18)	89.62 (9.66)	0.595
Role function			
Before the first cycle of chemotherapy	88.41 (16.80)	84.23 (17.54)	0.230
Before the second cycle of chemotherapy	91.29 (12.70)	91.89 (12.80)	0.770
Before the third cycle of chemotherapy	85.93 (15.06)	93.33 (11.58)	0.023
Cognitive function			
Before the first cycle of chemotherapy	87.68 (16.27)	90.09 (12.08)	0.712
Before the second cycle of chemotherapy	91.29 (12.18)	92.34 (14.48)	0.392
Before the third cycle of chemotherapy	88.52 (13.68)	93.33 (11.58)	0.093
Emotional function			
Before the first cycle of chemotherapy	87.32 (13.69)	80.86 (17.88)	0.086
Before the second cycle of chemotherapy	88.07 (14.86)	88.96 (15.72)	0.837
Before the third cycle of chemotherapy	87.04 (13.36)	90.95 (11.32)	0.244
Social function			
Before the first cycle of chemotherapy	76.45 (24.24)	77.93 (15.74)	0.942
Before the second cycle of chemotherapy	83.33 (17.24)	84.23 (21.50)	0.518
Before the third cycle of chemotherapy	86.67 (17.26)	87.62 (16.83)	0.839
Symptom dimensions			
Fatigue			
Before the first cycle of chemotherapy	19.32 (14.52)	23.72 (13.65)	0.175
Before the second cycle of chemotherapy	21.26 (21.95)	15.77 (13.19)	0.443
Before the third cycle of chemotherapy	16.30 (13.10)	11.90 (12.93)	0.118
Pain			
Before the first cycle of chemotherapy	15.94 (20.17)	10.81 (17.22)	0.213
Before the second cycle of chemotherapy	17.75 (25.68)	6.76 (13.87)	0.021
Before the third cycle of chemotherapy	10.00 (13.94)	8.10 (10.97)	0.648
Nausea and vomiting			
Before the first cycle of chemotherapy	5.07 (10.46)	4.05 (9.13)	0.715
Before the second cycle of chemotherapy	22.46 (24.14)	4.05 (9.13)	<0.001
Before the third cycle of chemotherapy	27.04 (29.15)	0.00 (0.00)	<0.001
Dyspnea			
Before the first cycle of chemotherapy	21.74 (20.14)	27.03 (17.28)	0.169
Before the second cycle of chemotherapy	19.57 (24.92)	14.41 (16.74)	0.510
Before the third cycle of chemotherapy	17.78 (18.26)	13.33 (16.57)	0.291
Insomnia			
Before the first cycle of chemotherapy	26.81 (22.90)	22.52 (20.87)	0.412
Before the second cycle of chemotherapy	20.30 (26.74)	10.81 (15.82)	0.130
Before the third cycle of chemotherapy	20.00 (22.92)	14.29 (16.74)	0.351
Appetite loss			
Before the first cycle of chemotherapy	10.87 (17.29)	8.11 (16.49)	0.390
Before the second cycle of chemotherapy	22.46 (24.40)	9.91 (15.45)	0.010
Before the third cycle of chemotherapy	22.22 (18.80)	6.67 (13.53)	<0.001
Constipation			
Before the first cycle of chemotherapy	8.70 (16.38)	6.31 (13.24)	0.556
Before the second cycle of chemotherapy	23.91 (24.00)	6.31 (13.24)	<0.001
Before the third cycle of chemotherapy	28.15 (22.42)	0.95 (5.63)	<0.001
Diarrhea			
Before the first cycle of chemotherapy	7.97 (14.38)	5.41 (14.73)	0.266
Before the second cycle of chemotherapy	9.42 (22.95)	1.80 (7.64)	0.056
Before the third cycle of chemotherapy	15.56 (16.82)	2.86 (9.47)	<0.001
Financial difficulties			
Before the first cycle of chemotherapy	30.43 (32.07)	26.13 (32.52)	0.455
Before the second cycle of chemotherapy	23.91 (31.94)	19.82 (30.89)	0.443
Before the third cycle of chemotherapy	20.00 (26.01)	18.10 (28.40)	0.491

Abbreviations: SD, standard deviation.

**FIGURE 2:**
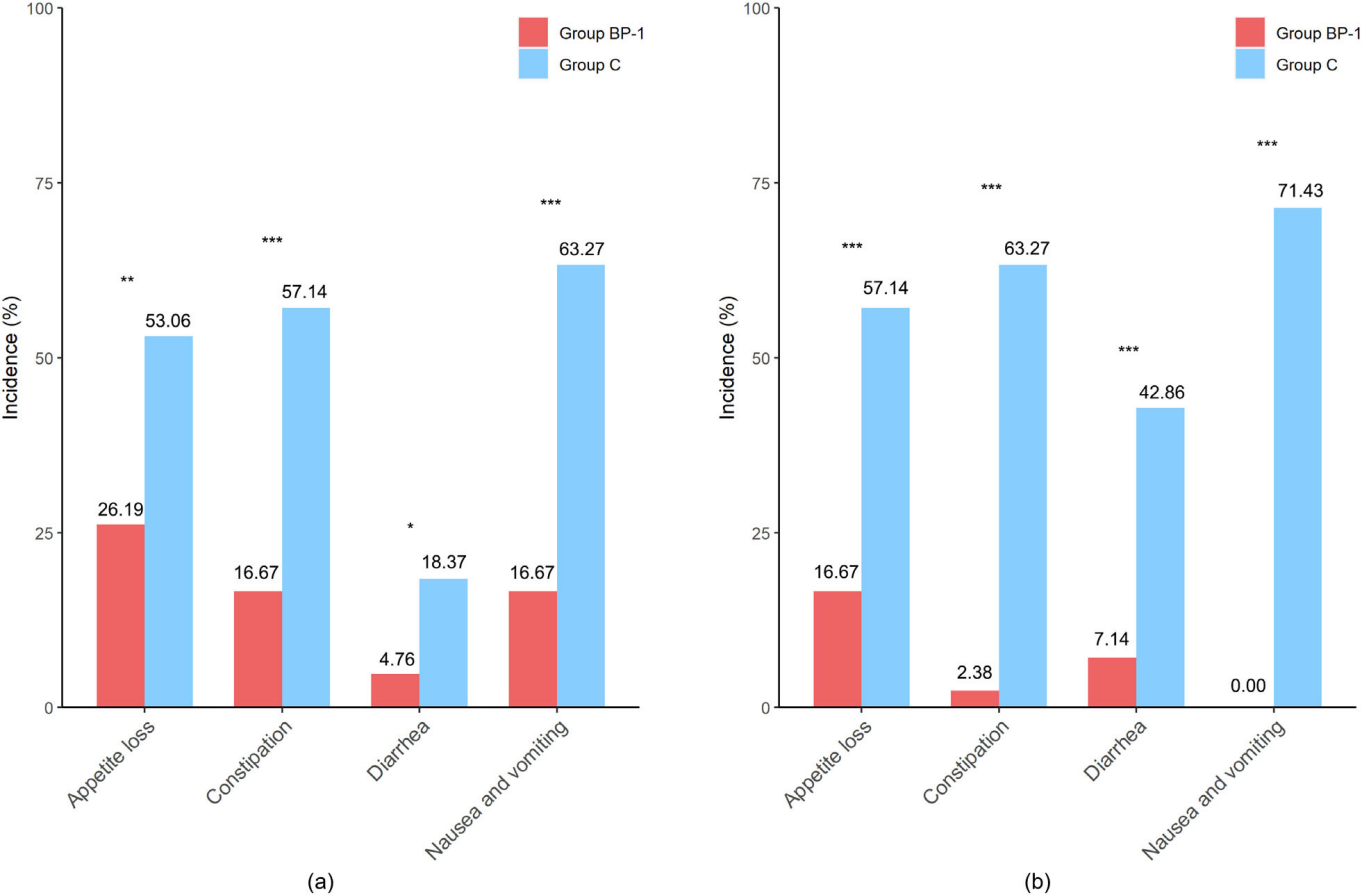
The prevalence of chemotherapy‐related gastrointestinal reactions between group BP‐1 and group C. (a) The incidence of chemotherapy‐related gastrointestinal reactions during the first cycle of chemotherapy. (b) The incidence of chemotherapy‐related gastrointestinal reactions during the second cycle of chemotherapy; *p < 0.05; **p < 0.01; ***p < 0.001.

After one cycle of chemotherapy and compound probiotic supplement/placebo treatment, the scores of the two groups (group BP‐1 vs. group C) showed a statistically significant difference in the following dimensions: overall quality of life (75.45 ± 15.46 vs. 61.55 ± 19.12, *p* = 0.001), pain (6.76 ± 13.87 vs. 17.75 ± 25.68, *p* = 0.021), nausea and vomiting (4.05 ± 9.13 vs. 22.46 ± 24.14, *p* < 0.001), appetite loss (9.91 ± 15.45 vs. 22.46 ± 24.40, *p* = 0.010), and constipation (6.31 ± 13.24 vs. 23.91 ± 24.00, *p* < 0.001). The incidence of nausea and vomiting, appetite loss, constipation, and diarrhea in group BP‐1 was 16.67%, 26.19%, 16.67%, and 4.76%, respectively, significantly lower than that of the group C: 63.27%, 53.06%, 57.14%, and 18.37%, respectively (*p* < 0.05) (Table 2, Figure 2a).

After 2 cycles of the study treatment, there was a significant difference in the dimensions, including overall quality of life (76.90 ± 18.31 vs. 58.89 ± 17.17, *p* < 0.001), role function (93.33 ± 11.58 vs. 85.93 ± 15.06, *p* = 0.023), nausea and vomiting (0.00 ± 0.00 vs. 27.04 ± 29.15, *p* < 0.001), appetite loss (6.67 ± 13.53 vs. 22.22 ± 18.80, *p* < 0.001), constipation (0.95 ± 5.63 vs. 28.15 ± 22.42, *p* < 0.001) and diarrhea (2.86 ± 9.47 vs. 15.56 ± 16.82, *p* < 0.001) between the group BP‐1 and group C. The incidence of nausea and vomiting, appetite loss, constipation, and diarrhea in group BP‐1 was significantly lower than in group C (0% vs. 71.43%, 16.67% vs. 57.14%, 2.38% vs. 63.27%, and 7.14% vs. 42.86%, respectively, *p* < 0.001) (Table 2, Figure 2b).

### Adverse events

No grade>3 adverse events were observed. There were no differences in adverse reactions between the two groups, except for the incidence of gastrointestinal reactions. In particular, the incidence of nausea and vomiting, constipation, anorexia, and diarrhea were lower in group BP‐1 than in group C (Table 3).

**TABLE 3:** Adverse events.

Toxicity	Grade 1		Grade 2		Grade 3		*p*‐value
	Group BP‐1 *N* (%)	Group C *N* (%)	Group BP‐1 *N* (%)	Group C *N* (%)	Group BP‐1 *N* (%)	Group C *N* (%)	
Neutropenia	10 (23.81)	14 (28.57)	6 (14.29)	9 (18.37)	1 (2.38)	2 (4.08)	0.778
Anemia	8 (19.05)	15 (30.61)	8 (19.05)	4 (8.16)	0 (0.00)	1 (2.04)	0.209
Thrombocytopenia	9 (21.43)	7 (14.29)	1 (2.38)	2 (4.08)	‐	‐	0.624
Sensory neuropathy	5 (11.90)	6 (12.24)	1 (2.38)	3 (6.12)	1 (2.38)	0 (0.00)	0.509
Nausea and vomiting	5 (11.90)	17 (34.69)	2 (4.76)	16 (32.65)	0 (0.00)	2 (4.08)	<0.001
Anorexia	10 (23.81)	22 (44.90)	1 (2.38)	6 (12.24)	‐	‐	0.007
Constipation	7 (16.67)	26 (53.06)	0 (0.00)	5 (10.20)	‐	‐	<0.001
Diarrhea	3 (7.14)	13 (26.53)	0 (0.00)	7 (14.29)	0 (0.00)	1 (2.04)	<0.001
Fatigue	19 (45.24)	21 (42.86)	7 (16.67)	10 (20.41)	0 (0.00)	2 (4.08)	0.413
Pain	3 (7.14)	7 (14.29)	2 (4.76)	6 (12.24)	‐	‐	0.201
Dyspnea	3 (7.14)	6 (12.24)	0 (0.00)	3 (6.12)	‐	‐	0.098
Insomnia	5 (11.90)	5 (10.20)	3 (7.14)	5 (10.20)	‐	‐	0.857
Dizziness	3 (7.14)	5 (10.20)	1 (2.38)	0 (0.00)	‐	‐	0.408
Alopecia	4 (9.52)	3 (6.12)	2 (4.76)	2 (4.08)	‐	‐	0.816

## DISCUSSION

Our study demonstrated that compound probiotic supplements can improve the quality of life and relieve platinum‐based doublet chemotherapy‐induced gastrointestinal adverse reactions for lung cancer patients undergoing chemotherapy. Previous clinical studies have also indicated that probiotics may ameliorate chemotherapy‐induced adverse effects. Jiang et al. found that a probiotic combination (*Bifidobacterium longum*, *Lactobacillus lactis*, and *Enterococcus faecium*) can ameliorate the severity of oral mucositis via gut microbiota modulation for nasopharyngeal carcinoma patients who were undergoing concurrent radiochemotherapy.^28^ Probiotic combinations containing *Bifidobacterium infants*, *Lactobacillus acidophilus*, *Enterococcus faecalis*, and *Bacillus cereus* have also been shown to be effective in attenuating chemotherapy‐related gastrointestinal complications, especially diarrhea for colorectal cancer patients who were undergoing postoperative chemotherapy.^22^ As for lung cancer patients receiving platinum‐based doublet chemotherapy, *Clostridium butyricum* can relieve chemotherapy‐related diarrhea.^25^ However, clinical studies exploring the usage of probiotics to mitigate chemotherapy‐related adverse effects have predominantly concentrated on colorectal cancer and head and neck carcinoma, with fewer studies targeting lung cancer patients. In addition, there has been limited exploration regarding whether combination probiotic preparations containing *Lactobacillus* and *Bifidobacterium* can improve chemotherapy‐related adverse effects for lung cancer patients. Our study provides preliminary evidence favoring the potential benefits of compound probiotic supplements to ameliorate chemotherapy‐related adverse effects and the possibility of compound probiotic clinical application in managing chemotherapy‐related complications among lung cancer patients.

It is our inaugural endeavor to improve the quality of life for lung cancer patients who are undergoing chemotherapy through compound probiotic supplements. In this report, after two cycles of compound probiotic supplement/placebo treatment along with platinum‐based doublet chemotherapy, a significant difference in some questionnaire dimensions was shown between the two groups. Most of all, the overall quality of life score in group BP‐1 was significantly better than that in group C, and so was the score of role function. In other words, patients in group BP‐1 maintained a relatively good quality of life during the chemotherapy course, which was aggravated in group C. Moreover, the prevalence of nausea and vomiting, appetite loss, constipation, and diarrhea in group BP‐1 was significantly lower than in group C. The above results imply that the adverse effects caused by chemotherapy may worsen the quality of life. A randomized controlled trial reported a similar situation: the quality of life analysis of KEYNOTE‐024 showed significantly higher scores in the QLQ‐30 questionnaire for nausea and vomiting, constipation, and diarrhea in the chemotherapy group.^29^ The symptom control and quality of life investigation conducted in the LUX‐Lung 3 trial performed the QLQ‐30 questionnaire, revealing that 63% of patients in the chemotherapy group experienced nausea and vomiting, and 24% of patients experienced diarrhea following pemetrexed plus cisplatin treatment.^30^ Our study observed that the prevalence of nausea and vomiting in group C was 63.27% and 71.43% before the second and third cycle of chemotherapy, respectively; the incidence of diarrhea was 18.37% and 42.86%, respectively, which were close to the findings from the LUX‐Lung 3 trial. Meanwhile, the incidence and the QLQ‐30 questionnaire scores of diarrhea and vomiting in the BP‐1 group were lower than those reported in the LUX‐Lung 3 trial. These findings indicate that the compound probiotics supplements can relieve gastrointestinal side effects; for example, diarrhea and vomiting, thereby maintaining the patient's quality of life during chemotherapy.

How do the probiotics work on improving the quality of life in patients receiving chemotherapy?

Chemotherapeutic agents can disturb the composition and diversity of the gut microbiota, correlated with adverse effects such as diarrhea, appetite loss, etc.^5^, ^6^, ^31^ Considering the close connection between the composition of gut microbiota and short‐chain fatty acids (SCFAs) production,^32^ it is plausible to hypothesize that chemotherapy‐related gastrointestinal reactions may be attributed to a decline in SCFA levels resulting from an imbalance in gut microbiota post‐chemotherapy. SCFAs have been demonstrated to attenuate chemotherapy‐related toxicities due to their anti‐inflammatory, antioxidant, and protective characteristics.^32^ SCFAs can also be against chemotherapy‐induced intestinal injury via immunoregulation, promoting crypt cell proliferation and maintaining epithelial integrity.^32^, ^33^ In addition, postoperative chemotherapy may lead to a decline in gut phylum Firmicutes levels for colorectal cancer patients,^22^ which are known to be an important source of SCFAs.^34^ *Bifidobacterium* and *Lactobacillus* are SCFA‐producing microbiota; supplementation with compound probiotics can potentially reduce chemotherapy‐related gastrointestinal adverse events for lung cancer patients by restoring SCFA levels and thus improving quality of life. Our aim is to validate this in future studies.

It is a pity that the sample size of this study was limited after 19 patients were excluded from the final statistical analysis. Further exploration of the variation of gut microbiota and SCFA levels are needed to elucidate the potential mechanisms of compound probiotic supplements to ameliorate chemotherapy‐related adverse effects. In addition, many patients with early‐stage lung cancer were included in our study, and our future studies will be focused on patients with advanced lung cancer. Our study observed that compound probiotic supplements could effectively alleviate gastrointestinal adverse events. We still need to explore whether compound probiotic supplements can improve other chemotherapy‐related adverse reactions and improve the quality of life of cancer patients in future studies. Probiotic agents have been reported to provide a survival benefit for lung cancer patients treated with ICIs.^35^ Our study focused mainly on the management of chemotherapy‐related adverse effects and neglected the observation of treatment efficacy, which will be further explored in a subsequent study. However, this study is the first step in evaluating compound probiotic supplement intervention in improving the quality of life and relieving the symptoms of patients suffering from the adverse effects of chemotherapy. We have found a positive trend from the current study. We also plan further clinical trials to provide more robust evidence to confirm the advantages of compound probiotic supplements to lung cancer patients undergoing chemotherapy.

In conclusion, oral compound probiotic supplements can improve the quality of life and relieve chemotherapy‐related gastrointestinal adverse events for lung cancer patients receiving platinum‐based chemotherapy.

## AUTHOR CONTRIBUTIONS

All authors had full access to the data in the study and take responsibility for the integrity of the data and the accuracy of the data analysis. *Conceptualization*, Yu Sun and Jiang Zhu; *Data curation*, Jialong Han, Ping Chen, and Ke Xie; *Formal analysis*, Hao Wei, Zhiying Yue, and Jialong Han; *Funding acquisition*, Yu Sun and Jiang Zhu; *Investigation*, Jialong Han, Ping Chen, and Ke Xie; *Methodology*, Yu Sun and Jiang Zhu; *Project administration*, Yu Sun and Jiang Zhu; *Resources*, Jialong Han, Ping Chen, and Ke Xie; *Software*, Hao Wei and Zhiying Yue; *Supervision*, Yu Sun and Jiang Zhu; *Validation*, Yu Sun and Jiang Zhu; *Visualization*, Hao Wei; *Writing – original draft preparation*, Hao Wei, Zhiying Yue, and Jialong Han; *Writing – Review and editing*, Jiang Zhu.

## CONFLICT OF INTEREST STATEMENT

The Authors declare that there is no conflict of interest.
